# Impact of ongoing intravenous thrombolysis until completion of endovascular treatment in large vessel occlusion stroke patients

**DOI:** 10.3389/fneur.2023.1231530

**Published:** 2023-08-04

**Authors:** Johannes Wischmann, Cauchy Pradhan, Hanna Zimmermann, Linus Keidel, Steffen Tiedt, Konstantinos Dimitriadis, Thomas Liebig, Günter Höglinger, Lars Kellert

**Affiliations:** ^1^Department of Neurology, LMU University Hospital, LMU Munich, Munich, Germany; ^2^German Center for Vertigo and Balance Disorders, University Hospital, LMU Munich, Munich, Germany; ^3^Institute of Neuroradiology, LMU University Hospital, LMU Munich, Munich, Germany; ^4^Institute for Stroke and Dementia Research (ISD), LMU University Hospital, LMU Munich, Munich, Germany

**Keywords:** acute ischemic stroke, intravenous thrombolysis, thrombectomy, intravenous thrombolysis timing, ongoing IVT

## Abstract

**Background:**

Recent studies have implied that ongoing intravenous thrombolysis (IVT) during endovascular treatment (ET) improves functional outcomes in patients who have undergone stroke caused by a large vessel occlusion (LVO). In this study, we investigated the effect of ongoing IVT until completion of ET on procedure duration, first-pass thrombectomy rate, and periprocedural complications.

**Methods:**

We analyzed patients from the German Stroke Registry-Endovascular Treatment dataset, collected between June 2015 and December 2021. Primary outcomes were modified Rankin Scale (mRS) score after 3 months and achievement of a Thrombolysis In Cerebral Infarction (TICI) score of 2b-3. Secondary parameters included ET duration, first-pass thrombectomy, and periprocedural complications.

**Results:**

Of the 13,082 patients in the dataset, 1,639 met the study inclusion criteria. A total of *n* = 317 patients (19.3%) underwent ongoing IVT until completion of ET, while IVT was completed prior to ET in 1,322 patients (80.7%). Ongoing IVT was associated with higher rates of achievement of an mRS score of 0–2 (or a back-to-baseline) after 3 months [odds ratio (OR) 1.53; 95% confidence interval (CI) 1.08–2.17]. Furthermore, ongoing IVT was predictive of achievement of a TICI score of 2b-3 (OR 1.37; 95% CI 1.03–1.83) and of first-pass thrombectomy (OR 2.07; 95% CI 1.51–2.84), while reducing the rate of peri-interventional complications (OR 0.64; 95% CI 0.44–0.94) and reducing ET duration by 24 min [β = −24.35; 95% CI −32.92–(−15.79)].

**Conclusion:**

Our findings suggest that ongoing IVT until ET completion has a favorable impact on both clinical and angiographic outcomes, as well as on periprocedural conditions, regardless of the overall time intervals involved. Therefore, rapid ET after IVT should be sought in order to take advantage of the additive effect of ongoing IVT during ET. Future studies should consider IVT timing in the context of ET as a potential confounder and treatment target.

## Introduction

Intravenous thrombolysis (IVT) and endovascular treatment (ET) are the standard therapies for patients with acute ischemic stroke (AIS) due to a large vessel occlusion (LVO). If patients are eligible for both treatments, current guidelines recommend IVT prior to ET (bridging therapy) (1–3). Both treatments are time-sensitive and rapid initiation after symptom onset (SO) is recommended, as delayed flow restoration (FLR) clearly correlates with poor functional outcome (4). However, little is known about the impact of the timing of IVT at specific time points relative to ET on functional and angiographic outcome. The CHOICE trial demonstrated an improved clinical outcome in AIS patients receiving adjunct intra-arterial thrombolysis (IAT) after successful FLR (5). Possible explanations for the observed results included improved microcirculation and thrombolysis of more distal vessel occlusions, emphasizing a synergistic effect of IVT when applied at FLR. Regarding the use of adjunct IVT at FLR, no randomized controlled trials are available. To investigate the issue, two studies were conducted using data from the German Stroke Registry-Endovascular Treatment (GSR-ET). The first study involved 1,303 patients and found that ongoing intravenous thrombolysis (IVT) upon FLR was associated with better functional outcomes after 90 days in patients with a Thrombolysis In Cerebral Infarction score 65 (TICI)-Score of 2b-3, compared to those who had finished IVT (6). This suggests that the results of the CHOICE trial can be applied in a real-world setting. The second study used a matched case–control analysis and found that simultaneous administration of IVT during ET (with a minimum overlap of 10 min) was beneficial for patients with large-vessel occlusion (LVO) stroke and a premodified Rankin Scale (pmRS) score of 0–3 (7).

For this analysis, we first aimed to validate those findings in a larger cohort from the GSR-ET, emphasizing a possible synergistic beneficial effect of IVT during ET on clinical outcome. Second, we aimed to expand the focus on the impact of simultaneous administration of IVT during ET to ET-related technical parameters, including ET duration, rates of first-pass thrombectomy, and peri-interventional complications.

## Materials and methods

### Study cohort

Data were obtained from the German Stroke Registry-Endovascular Treatment (GSR-ET), which has been previously described in detail (4, 8). In brief, the GSR-ET (ClinicalTrials.gov identifier NCT03356392) is a prospective multicenter registry aiming to systematically assess outcomes, safety, and peri-interventional parameters in adult LVO AIS patients who are intended to be treated with ET. Patients were enrolled consecutively at 25 centers in Germany. Clinical decisions for treatment with ET and/or IVT were based on current national and international guidelines (1, 2). IVT was carried out by administering alteplase at 0.9 mg/kg body weight over 1 h (10% bolus), as recommended in national and international guidelines. Tenecteplase was not used in any participating center. Clinical and radiological data on all patients were rated and assessed by local neurologists and neuroradiologists on each side. Stroke severity and functional independence before and after the qualifying stroke were rated using the National Institutes of Health Stroke Scale (NIHSS) and the (pre)-modified Rankin Scale (pmRS), respectively. In patients with unknown SO, the median time point between “last seen well” and “time of recognition” was considered as the time of SO for further calculations. The type and side of vessel occlusion were determined using computed tomography angiography (CTA), magnetic resonance angiography (MRA), or digital subtraction angiography (DSA). Early radiological signs of cerebral infarction were assessed using the Alberta Stroke Program Early CT Score (ASPECTS). Degree of reperfusion at final DSA was rated using the mTICI scale. Follow-up parameters included predefined peri-interventional complications [vasospasm, clot migration/embolization, vessel dissections/perforations, intracranial hemorrhage (ICH) after 24 h, and malignant middle cerebral artery infarction] and mRS after 90 days (assessed either by on-site visit or by telephone interview). ICH was defined as any intracranial hemorrhage observed in postinterventional CT imaging after 24 h, irrespective of the presence of new clinical symptoms. All data were checked for plausibility, integrity, and completeness, using a computed standardized protocol. In cases of inconsistent data, queries were sent to the corresponding team to request reassessment.

### Definition of inclusion criteria

**Figure 1:**
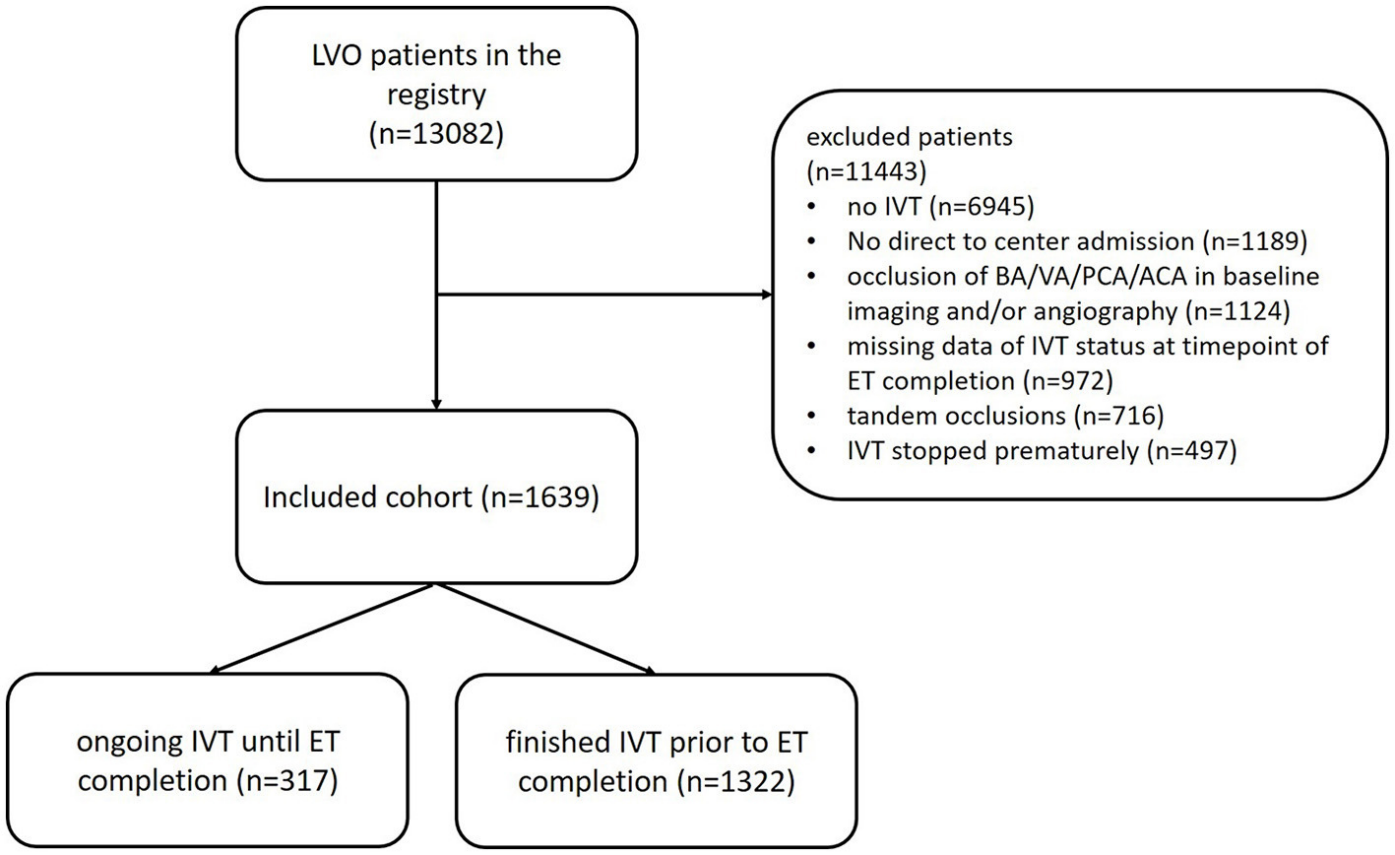
Flowchart illustrating the selection and exclusion of patients for final analysis. LVO, large vessel occlusion; IVT, intravenous thrombolysis; BA, basilar artery; VA, vertebral artery; PCA, posterior cerebral artery; ACA, anterior cerebral artery.

Source data were collected, including patients registered between June 2015 and December 2021. We included all anterior circulation LVO AIS patients who received both IVT and ET. Type of vessel occlusion was assessed, considering baseline CTA, MRA, and angiography. Patients for whom IVT was prematurely stopped prior to ET were excluded. As prolonged time intervals and stenting during ET are more frequent in posterior circulation stroke and tandem lesions (9, 10), such patients were excluded, avoiding possible confounders and increasing the homogeneity of the cohort. Only patients admitted directly to an ET center were considered for final analysis in order to avoid major time interval differences. Ongoing IVT until completion of ET was assumed at a time interval between the start of IVT and the time point of the best achievable FLR of ≤ 60 min. Patients with missing time intervals for evaluation of IVT status at FLR were excluded. After application of the inclusion criteria, *n* = 1,639 patients were considered for further analysis (Figure 1).

### Primary and secondary outcomes

The mRS score at 90 days and the mTICI score were defined as the primary outcome parameters. An mRS of 0–2 or back-to-baseline pmRS was considered to represent a good clinical outcome, and a TICI score of 2b-3 was defined as a good angiographic outcome. ET duration (as defined by time from groin puncture to FLR), first-pass thrombectomy, and peri-interventional complications [vasospasm, clot migration/embolization, vessel dissections/perforations, intracranial hemorrhage (ICH) after 24 h, and malignant middle cerebral artery infarction] were defined as secondary outcome parameters. First-pass thrombectomy was defined as vessel reperfusion achieved through a single retrieval attempt, resulting in a TICI score of 2b-3 (11).

### Statistical analysis

Univariate analysis was carried out, with each variable expressed in the form of mean ± standard deviation (SD), median [interquartile range (IQR)], or counts and percentages, as applicable. Variables were checked for normal distribution using the Kolmogorov–Smirnov test. Differences between the two groups were evaluated using the Mann–Whitney *U*-test and the chi-squared test where appropriate. Binary and multiple linear regression analyses were conducted for primary and secondary outcome parameters, adjusting for potential confounders that were either statistically significant in the univariate analysis or known to be confounders for outcome parameters. Shift in mRS at 90 days was estimated using ordinal regression analysis. A p-value < 0.05 was considered to represent statistical significance. SPSS version 26 for Windows (IBM Corp, Armonk, NY) was employed for all statistical analyses.

## Results

### Univariate analysis

Of the *n* = 1,639 patients considered for analysis, *n* = 317 (19.3%) underwent ongoing IVT until ET completion, while in *n* = 1,322 patients (80.7%), IVT was completed (Table 1).

**Table 1:** Univariate analysis comparing patients who underwent ongoing IVT and those who had completed IVT at the time point of ET completion.

	**Ongoing IVT until ET completion (*n* = 317; 19.3%)**	**Finished IVT prior to ET completion (*n* = 1,322; 80.7%)**	***P***	**Included patients, *n* (%)**
**Baseline characteristics**				
Age (years), median (IQR)	76 (64–82)	76 (66–84)	0.13	1,637 (99.9)
Female sex, *n* (%)	168 (53.0)	708 (53.6)	0.85	1,638 (99.9)
NIHSS, median (IQR)	14 (8–18)	14 (10–18)	0.24	1,630 (99.5)
pmRS, median (IQR)	0 (0–0)	0 (0–0)	0.63	1,624 (99.1)
ASPECTS	9 (8–10)	9 (8–10)	0.23	1,585 (96.7)
**Time intervals (minutes)**				
SO to admission, median (IQR)	69 (53–110)	73 (53–120)	0.28	1,575 (96.1)
Admission to groin puncture, median (IQR)	57 (46–69)	82 (65–107)	< 0.05	1,627 (99.3)
Groin puncture to FLR, median (IQR)	23 (16–30)	43 (29–65)	< 0.05	1,627 (99.3)
Admission to FLR, median (IQR)	80 (71–93)	132 (107–165)	< 0.05	1,639 (100)
IVT to FLR, median (IQR)	50 (41–55)	101 (79–131)	< 0.05	1,639 (100)
SO to IVT, median (IQR)	105 (81–150)	104 (78–155)	0.51	1,584 (96.6)
**Type and side of vessel occlusion**, ***n*** **(%)**				
Extracranial carotid artery	5 (1.6)	36 (2.7)	0.24	
Intracranial carotid artery (excluding T)	12 (3.8)	54 (4.1)	0.81	
Carotid-T	35 (11.0)	200 (15.1)	0.07	
M1, proximal	123 (38.8)	418 (31.6)	< 0.05	
M1, distal	79 (24.9)	315 (23.8)	0.68	
M2	79 (24.9)	454 (34.3)	< 0.05	
Left-sided vessel occlusion	167 (53.0)	672 (51.3)	0.58	1,626 (99.2)
**Endovascular treatment**				
Retrieval attempts, median (IQR)	1 (1–2)	2 (1–3)	< 0.05	1,570 (95.8)
First-pass thrombectomy, *n* (%)	210 (69.8)	574 (46.1)	< 0.05	1,545 (94.3)
IA medication, *n* (%)	46 (14.7)	200 (15.2)	0.82	1,627 (99.3)
IAT, *n* (%)	7 (2.2)	46 (3.5)	0.25	
TICI 2b-3, *n* (%)	308 (97.2)	1,224 (93.2)	< 0.05	1,630 (99.5)
**Risk factors**				
Hypertension, *n* (%)	221 (70.8)	969 (73.7)	0.31	1,627 (99.3)
Diabetes, *n* (%)	54 (17.3)	242 (18.4)	0.64	1,629 (99.4)
Dyslipidemia, *n* (%)	142 (45.2)	528 (30.3)	0.11	1,625 (99.1)
Atrial fibrillation, *n* (%)	111 (35.5)	488 (37.1)	0.58	1,627 (99.3)
Smoking, *n* (%)	81 (26.4)	304 (24.3)	0.45	1,558 (95.1)
Systolic BP (mmHg), mean ± SD	149.4 ± 26.8	152.8 ± 27.0	< 0.05	1,472 (89.8)
Diastolic BP (mmHg), mean ± SD	83.0 ± 17.7	83.3 ± 17.3	0.49	1,463 (89.3)
HR (bpm), mean ± SD	81.9 ± 19.2	81.2 ± 19.2	0.39	1,457 (88.9)
Antiplatelet therapy, *n* (%)	100 (32.6)	459 (35.1)	0.39	1,613 (98.4)
OAC therapy, *n* (%)	19 (6.2)	56 (4.3)	0.16	1,613 (98.4)
**Peri-interventional complications**				
Total, *n* (%)	49 (15.5)	311 (23.5)	< 0.05	
ICH after 24 h, *n* (%)	21 (6.6)	171 (12.9)	< 0.05	
Device malfunction, *n* (%)	0 (0.0)	2 (0.2)	0.49	
Vasospasm, *n* (%)	14 (4.4)	72 (5.4)	0.46	
Clot migration/embolization, *n* (%)	8 (2.5)	50 (3.8)	0.28	
Dissection or perforation, *n* (%)	9 (2.8)	40 (3.0)	0.86	
Malignant MCA infarction, *n* (%)	2 (0.6%)	37 (2.8)	< 0.05	
**Follow-up at 90 days**				
Good clinical outcome (mRS 0–2 or back to baseline), *n* (%)	183 (63.3)	586 (49.4)	< 0.05	1,476 (90.1)
mRS, median (IQR)	2 (0–4)	3 (1–5)	< 0.05	1,476 (90.1)

IVT, intravenous thrombolysis; ET, endovascular treatment; FLR, flow restoration; IQR, interquartile range; NIHSS, National Institutes of Health Stroke Scale; pmRS, premodified Rankin Scale; ASPECTS, Alberta Stroke Program Early CT Score; SO, symptom onset; IA, intra-arterial; IAT, intra-arterial thrombolysis; TICI, Thrombolysis In Cerebral Infarction score; BP, blood pressure; SD, standard deviation; OAC, oral anticoagulation; ICH, intracranial hemorrhage; MCA, middle cerebral artery; HR, heart rate.

Patients in both groups were comparable with respect to the main baseline parameters (age, sex, NIHSS and pmRS scores, and ASPECTS; see Table 1) and time from SO to admission (69 vs. 73 min; *p* = 0.28). However, the time interval from admission to groin puncture was shorter in patients who received ongoing IVT until ET completion (57 vs. 82 min; *p* < 0.05).

### Primary and secondary outcomes

**Figure 2:**
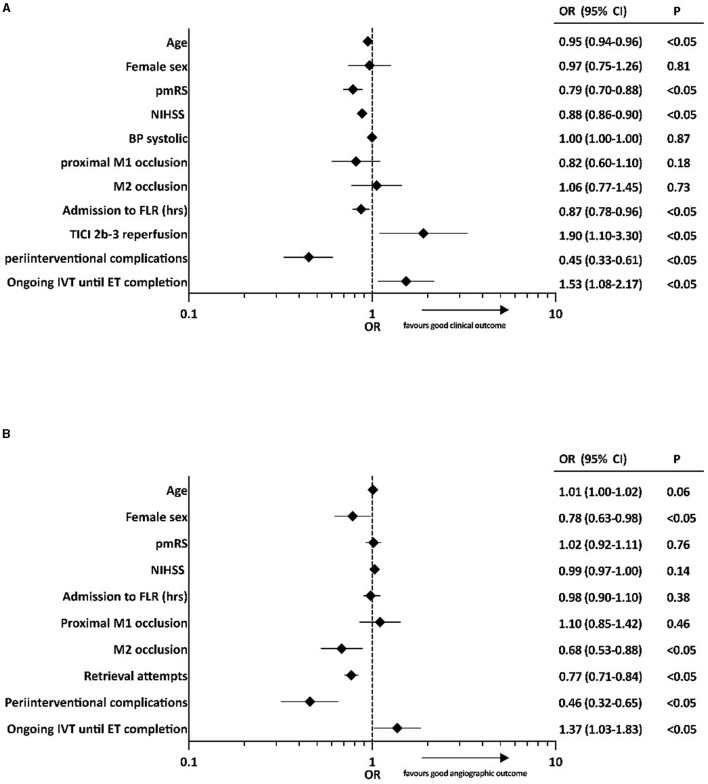
Binary logistic regression analyses of factors associated with **(A)** good clinical outcome (mRS 0–2 or back to baseline after 90 days) and **(B)** good angiographic outcome (TICI 2b-3). OR, odds ratio; CI, confidence interval; BP, blood pressure; pmRS, premodified Rankin Scale; NIHSS, National Institutes of Health Stroke Scale; FLR, flow restoration; TICI, Thrombolysis In Cerebral Infarction score; IVT, intravenous thrombolysis; ET, endovascular treatment.

**Figure 3:**
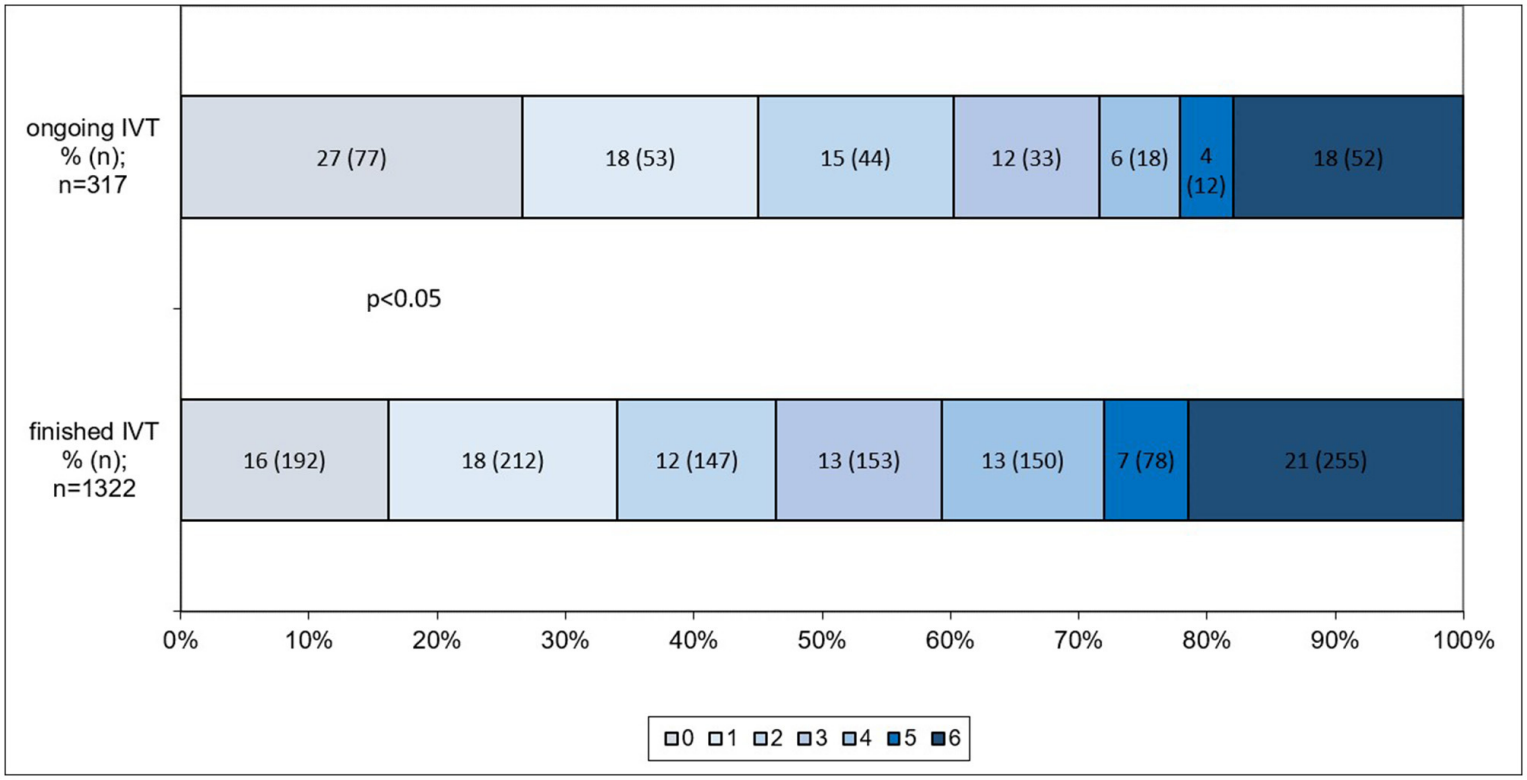
Distribution and shift analysis of mRS scores at 90 days of patients who received ongoing IVT and those who had finished IVT at the point of ET completion. A significant shift toward better functional outcomes was observed in patients who underwent ongoing IVT until ET completion, as indicated by ordinal regression analysis (OR 0.70; 95% CI 0.55–0.89; *p* < 0.05). mRS, modified Rankin Scale; IVT, intravenous thrombolysis; ET, endovascular treatment.

Based on univariate analysis, both a good clinical outcome (mRS 0–2 or back to baseline; 63.3 vs. 49.4%; *p* < 0.05) and a good angiographic outcome (97.2 vs. 93.2%; *p* < 0.05) were achieved more frequently in patients who received ongoing IVT until ET completion. Adjusting for potential confounders, ongoing IVT until ET completion was independently associated with a good clinical outcome (mRS 0–2 or back to baseline) after 3 months (odds ratio [OR] 1.53; 95% confidence interval [CI] 1.08–2.17; *p* < 0.05) (Figure 2A) and a good angiographic outcome at final angiography (OR 1.37; 95% CI 1.03–1.83; *p* < 0.05) (Figure 2B). Ordinal logistic regression analysis indicated a significant shift toward better functional outcomes after 90 days in patients who underwent ongoing IVT until ET completion (OR 0.70; 95% CI 0.55–0.89; *p* < 0.05; Figure 3).

Based on univariate analysis, ET duration, estimated as the time interval from groin puncture to FLR, was shorter (23 vs. 43 min; *p* < 0.05) and first-pass vessel reperfusion was achieved more frequently (69.8 vs. 46.1%; *p* < 0.05) in patients who received ongoing IVT until ET completion. The overall incidence of peri-interventional complications (vasospasm, clot migration/embolization, vessel dissections/perforations, ICH after 24 h, and malignant middle cerebral artery infarction) was lower in the ongoing-IVT group (15.5 vs. 23.5%; *p* < 0.05); additionally, ICH after 24 h in particular was less frequent in the ongoing-IVT group (6.6 vs. 12.9 %; *p* < 0.05).

**Figure 4:**
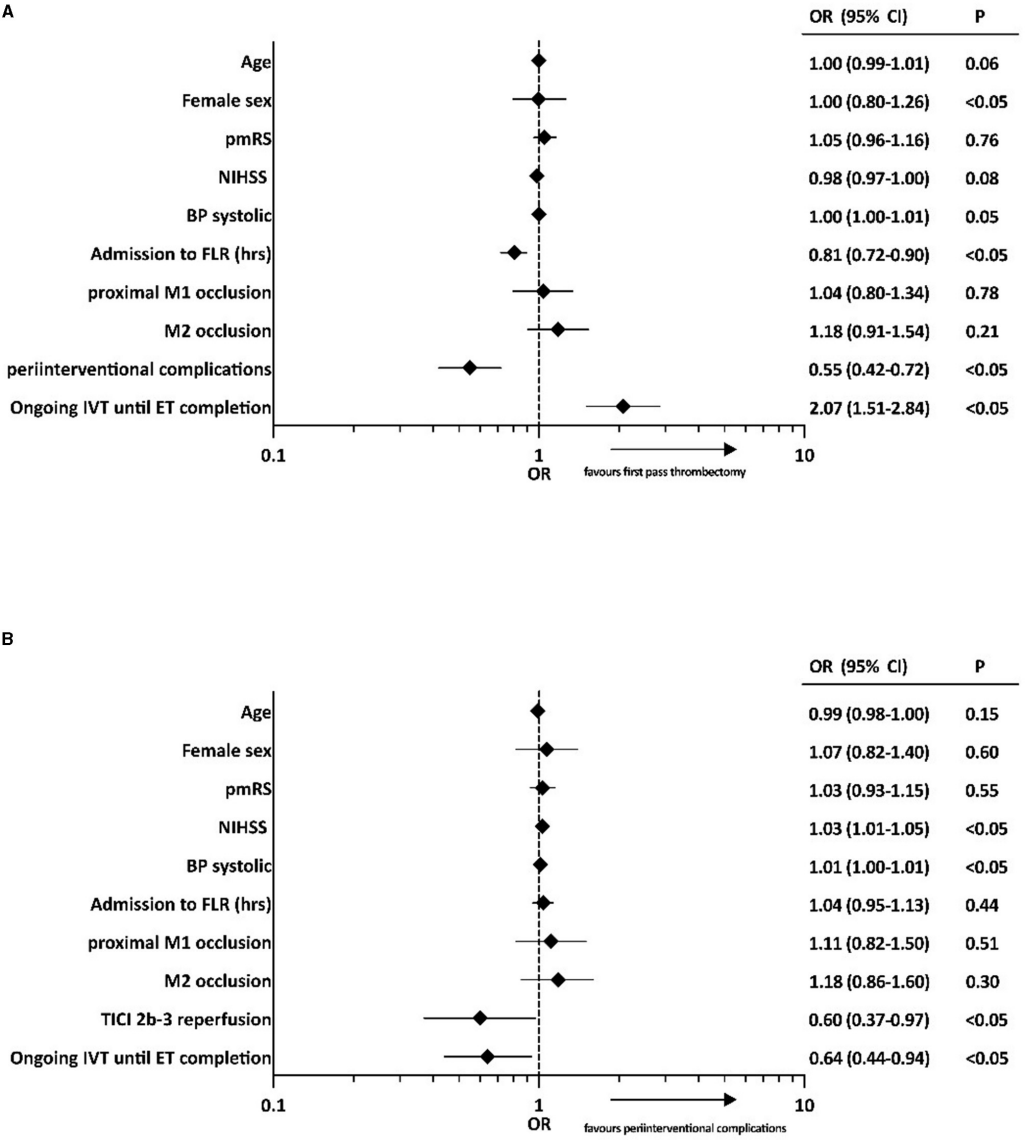
Binary logistic regression analyses of factors associated with **(A)** first-pass thrombectomy and **(B)** peri-interventional complications (vasospasm, clot migration/embolization, vessel dissections/perforations, ICH after 24 h, and malignant middle cerebral artery infarction). OR, odds ratio; CI, confidence interval; pmRS, premodified Rankin Scale; NIHSS, National Institutes of Health Stroke Scale; BP, blood pressure; FLR, flow restoration; TICI, Thrombolysis In Cerebral Infarction score; IVT, intravenous thrombolysis; ET, endovascular treatment.

Based on multiple linear regression analysis with adjustment for potential confounders, ongoing IVT until ET completion was associated with a reduction in ET duration of ~24 min (ß = −24.35; 95% CI −32.92–[−15.79]; *p* < 0.05) (Table 2). Furthermore, ongoing IVT until ET completion was independently associated with first-pass thrombectomy (OR 2.07; 95% CI 1.51–2.84; *p* < 0.05) (Figure 4A) and a reduced rate of peri-interventional complications (vasospasm, clot migration/embolization, vessel dissections/perforations, ICH after 24 h, and malignant middle cerebral artery infarction) based on the multivariate analysis (OR 0.64; 95% CI 0.44–0.94; *p* < 0.05; Figure 4B).

**Table 2:** Multiple linear regression analysis for ET duration (groin puncture to FLR [minutes])^*^.

**Variable**	**Coefficient (ß)**	**95% CI**	***P*-value**
Age (increase of 1 year)	0.01	−0.24–0.26	0.91
Female sex	7.03	0.39–13.66	< 0.05
pmRS (increase of 1 point)	0.83	−1.97–3.63	0.56
NIHSS at admission (increase of 1 point)	−0.05	−0.60–0.49	0.85
Systolic BP (increase of 1 mmHg)	−0.03	−0.15–0.09	0.63
Time from admission to groin puncture (increase of 1 min)	0.00	−0.04–0.04	0.94
Proximal M1 occlusion	−1.42	−9.01–6.17	0.71
M2 occlusion	4.77	−3.03–12.58	0.23
Retrieval attempts (increase by one attempt)	9.38	7.20–11.56	< 0.05
Peri-interventional complications	2.52	−5.44–10.48	0.54
Ongoing IVT until ET completion^*^	−24.35	−32.92–(−15.79)	< 0.05

^*^Adjusted for age, sex, pmRS, NIHSS, systolic BP, time interval from admission to groin puncture, proximal M1 occlusion, M2 occlusion, number of retrieval attempts, and ongoing IVT until ET completion. CI, confidence interval; pmRS, premodified Rankin Scale; NIHSS, National Institutes of Health Stroke Scale; BP, blood pressure; IVT, intravenous thrombolysis; ET, endovascular treatment. Peri-interventional complications include vasospasm, clot migration/embolization, vessel dissections/perforations, ICH after 24 h, and malignant middle cerebral artery infarction.

## Discussion

In this article, we have presented the impact of ongoing IVT vs. completed IVT at the point of ET completion in a large real-life dataset consisting of anterior circulation LVO patients. Our main results were as follows.

First, ongoing IVT at ET completion was associated with good clinical outcome (mRS 0–2 or back to baseline after 90 days; OR 1.53; 95% CI 1.08–2.17; *p* < 0.05), and we observed increased odds of achieving a better functional outcome by ~30% in the mRS shift analysis (OR 0.70; 95% CI 0.55–0.89; *p* < 0.05), which is in line with previous analyses (6, 7).

Second, patients who received ongoing IVT at ET completion were more likely to achieve successful reperfusion (mTICI 2b-3) at final angiography (OR 1.37; 95% CI 1.03–1.83; *p* < 0.05). Recent data indicate that IVT prior to ET is associated with higher reperfusion rates. However, data on the influence of IVT timing relative to ET on reperfusion rates are sparse; this is a new consideration and needs to be further elaborated on (12–14).

Third, we observed a reduction of procedure duration by ~24 min in patients who underwent ongoing IVT at ET completion (ß = −24.35; 95% CI −32.92–[−15.79]; *p* < 0.05). This results in accelerated reperfusion of the ischemic tissue, aligning with the observed improvement in functional outcome.

Fourth, ongoing IVT until ET completion turned out to be an independent predictor of first-pass thrombectomy (OR 2.07; 95%CI 1.51–2.84; *p* < 0.05), which is known to function as a predictor of favorable clinical outcome; this is likely linked with the reduced procedure duration (15). Considering that IVT has been shown to enhance first-pass thrombectomy rates, it is reasonable to also incorporate the discussion of IVT timing, although less is known about this aspect (16).

Finally, ongoing IVT until ET completion was associated with fewer peri-interventional complications (OR 0.64; 95% CI 0.44–0.94; *p* < 0.05), including vasospasm, clot migration/embolization, vessel dissections/perforations, ICH after 24 h, and malignant middle cerebral artery infarction, which most likely contributes to the improved functional outcomes observed in these patients.

Together, our findings may have substantial clinical implications. Not only are IVT and ET essentially additive, but they work particularly well together if IVT is still ongoing at the time of ET completion; this finding is consistent with recent analyses (6, 7). This highlights the need for continuous optimization of in-hospital processes in order to reach FLR within 60 min after IVT. This includes the ongoing development and refinement of standardized operating procedures for workflow and treatment algorithms and minimization of transit time from the emergency department to the angiosuite, as well as the establishment of dedicated multidisciplinary stroke teams (17). Moreover, methods for establishment of ongoing IVT at ET completion, e.g., by means of dynamic adaption of IVT flow rate or IVT splitting, represent an interesting target for further research. Furthermore, patients treated with tenecteplase prior to ET are more likely to have significant serum concentration of IVT at the time point of ET completion, due to its longer serum half-life, which may be considered when evaluating outcomes in those patients. While our data indicated that ongoing IVT at the point of ET completion is advantageous, it is crucial not to misinterpret this as a reason to withhold or postpone IVT. Rapid administration of IVT following stroke onset remains strongly associated with favorable functional outcomes (18).

This analysis was conducted on a large sample of prospectively assessed multicenter real-life data, which is a clear strength. Our analysis yielded very homogeneous results with respect to the generally positive effect of ongoing thrombolysis. The overall rate of both good clinical outcome and good angiographic outcome was comparable to that observed in recent trials, considering our inclusion criteria, which resulted in a sample comprising anterior circulation LVO stroke patients admitted directly to the center and receiving both IVT and ET (19, 20).

Our study has several limitations. Due to its observational nature, we are not able to draw conclusions on causality based on our results. As our assumption of ongoing IVT was based on retrospectively assessed time intervals, we also cannot exclude a possible selection bias. Furthermore, we cannot eliminate the possibility of residual confounders that we did not account for in our analysis, including factors such as the longer time interval from admission to groin puncture in the completed-IVT group. While key baseline characteristics were evenly distributed between the two groups, it is essential to acknowledge that our available observational data may not fully capture intrahospital time delays in particular. Consequently, we cannot rule out the potential for bias in the outcomes. Based on our inclusion criteria, we excluded patients for whom data were missing on either the timepoint of IVT administration or the timepoint of flow restoration. Additionally, we excluded patients with missing data regarding the receipt of IVT or a combination of these variables. Therefore, we cannot completely rule out the possibility of bias due to these missing data. Moreover, while the rate of local IAT during ET was comparable between both groups, we cannot provide information about the specific time point at which IAT was applied. Therefore, based on our data, we cannot distinguish a masked effect of local IAT application at the time of FLR from the possible effect of systemic IVT that was observed. Furthermore, it is important to note that, given our inclusion criteria, our study results are specifically applicable to patients directly admitted to a stroke center (the “mothership” model). In fact, achieving flow restoration through endovascular treatment within 60 min after administration of IVT would not be feasible in the majority of the “drip and ship” patients (21). Moreover, it is widely accepted that withholding or delaying IVT is not recommended, and this would in particular contradict the main principle of “drip and ship,” which has been shown to be an efficient concept in stroke care, particularly in rural areas (22, 23).

## Conclusion

We have demonstrated a beneficial impact of ongoing IVT until ET completion on both functional and angiographic outcomes in anterior circulation LVO patients admitted directly to a thrombectomy center. Ongoing IVT until ET completion may ease ET by means of facilitating first-pass thrombectomy and thus substantially reducing procedure times, as well as diminishing the rate of peri-interventional complications. This study highlights the need and potential for further research on the timing of IVT during ET.

## Data availability statement

The original contributions presented in the study are included in the article/supplementary material, further inquiries can be directed to the corresponding author.

## Ethics statement

The studies involving human participants were reviewed and approved by Ethics Committee of the LMU Munich (protocol: 689-15). Written informed consent for participation was not required for this study in accordance with the national legislation and the institutional requirements.

## Author contributions

JW and LKel conceptualized the study. JW, LKei, ST, and HZ collected the data. JW carried out the analysis and wrote the first draft of the manuscript. All authors commented on previous versions of the manuscript, read, and approved it in its final form.
